# Recycling Waste Cotton Cloths for the Isolation of Cellulose Nanocrystals: A Sustainable Approach

**DOI:** 10.3390/polym13040626

**Published:** 2021-02-19

**Authors:** Siti Hajar Mohamed, Md. Sohrab Hossain, Mohamad Haafiz Mohamad Kassim, Mardiana Idayu Ahmad, Fatehah Mohd Omar, Venugopal Balakrishnan, Muzafar Zulkifli, Ahmad Naim Ahmad Yahaya

**Affiliations:** 1 School of Industrial Technology, Universiti Sains Malaysia, Penang 11800, Malaysia; sitihajarmohamed95@gmail.com (S.H.M.); mhaafiz@usm.my (M.H.M.K.); mardianaidayu@usm.my (M.I.A.); 2 School of Civil Engineering, Universiti Sains Malaysia, Penang 14300, Malaysia; cefatehah@usm.my; 3 Institute for Research in Molecular Medicine (INFORMM), Universiti Sains Malaysia, Penang 11800, Malaysia; venugopal@usm.my; 4 Universiti Kuala Lumpur-Malaysian Institute of Chemical & Bioengineering Technology (UniKL-MICET), Melaka 78000, Malaysia; muzafar@unikl.edu.my

**Keywords:** waste cotton cloths, cellulose nanocrystals, solid waste management, sustainability, supercritical CO_2_

## Abstract

There is an interest in the sustainable utilization of waste cotton cloths because of their enormous volume of generation and high cellulose content. Waste cotton cloths generated are disposed of in a landfill, which causes environmental pollution and leads to the waste of useful resources. In the present study, cellulose nanocrystals (CNCs) were isolated from waste cotton cloths collected from a landfill. The waste cotton cloths collected from the landfill were sterilized and cleaned using supercritical CO_2_ (scCO_2_) technology. The cellulose was extracted from scCO_2_-treated waste cotton cloths using alkaline pulping and bleaching processes. Subsequently, the CNCs were isolated using the H_2_SO_4_ hydrolysis of cellulose. The isolated CNCs were analyzed to determine the morphological, chemical, thermal, and physical properties with various analytical methods, including attenuated total reflection-Fourier transform-infrared spectroscopy (ATR-FTIR), field-emission scanning electron microscopy (FE-SEM), energy-filtered transmission electron microscopy (EF-TEM), X-ray diffraction (XRD), thermogravimetric analysis (TGA), and differential scanning calorimetry (DSC). The results showed that the isolated CNCs had a needle-like structure with a length and diameter of 10–30 and 2–6 nm, respectively, and an aspect ratio of 5–15, respectively. Additionally, the isolated CNCs had a high crystallinity index with a good thermal stability. The findings of the present study revealed the potential of recycling waste cotton cloths to produce a value-added product.

## 1. Introduction

Sustainable waste management has become the main interest of the environmentalist in the 21st century, in order to minimize environmental pollution and greenhouse gas emissions. There is a growing concern regarding the generation of waste cotton cloths, with increasing cotton cloth production and a rising fast fashion demand in the textile and fashion industry [1,2,3]. It has been estimated that over 28 million tons of cotton cloths are produced and used globally [3,4]. About 20% of waste cotton cloths are either reused as they are or as wiping cloths, given to friends and relatives, and sold as pre-loved items, while others end up in open dumps at a landfill site or are incinerated [4,5,6]. It has been estimated that over 10 million tons of waste cotton cloths are disposed of every year globally [4,7,8]. These disposing methods cause environmental pollution and lead to a waste of useful resources. Although waste cotton cloths are biodegradable, the presence of chemical dye and pigment in clothes may pose a severe threat to the environment and aquatic ecosystem. 

Cellulose nanocrystals (CNCs) are natural polysaccharides that are generally isolated from biomass resources [9,10]. CNCs have been viewed as a promising material in many advance applications because of their distinct key features, including their abradant availability, unique optical properties, large surface area, high mechanical and thermal strength, environmentally friendly nature, and biodegradability [10,11]. Due to exhibiting many desirable properties, CNCs have been utilized in many advance applications, including biomedical engineering, antimicrobial agents, tissue engineering, drug delivery, biosensors, biocatalysts, and as an adsorbent [12]. Over the years, CNCs have been isolated from various lignocellulosic biomass, including plant cell walls [13], bamboo [14], wastepaper [15], cotton [16], cotton fabrics [11], algae [17], and bacteria [18]. However, waste cotton cloths contain over 90% cellulose. Therefore, the high cellulose content and abundant availability of waste cotton cloths have highlighted the potential to utilize waste cotton cloths as a promising raw material for synthesizing CNCs. The utilization of waste cotton cloths in CNC production would address many questions, including those related to (i) reducing the waste load disposed of in a landfill, (ii) minimizing the environmental pollution, and (iii) sustainably utilizing our waste resources [9,10,11].

Studies have been conducted on the isolation of CNCs from various cellulosic sources, including waste cotton cloths for use in various value-added products. Huang et al. [4] isolated CNCs from textile waste materials using sulfuric acid hydrolyses and a three-step oxidation process for utilizing them as a reinforcing agent of soy protein film. The study reported that the utilization of textile waste in CNCs is a promising approach for the sustainable utilization of waste materials to produce a value-added product. Shi et al. [19] isolated microcrystalline cellulose from waste cotton cloths using the hydrothermal method. It was found the isolated microcrystalline cellulose (MCC) had comparable chemo-mechanical and thermal properties to commercial microcrystalline cellulose. Wang et al. [9] reused waste cotton cloths for the extraction of CNCs using a mixed acid solution (H_2_SO_4_:HCl:deionized water = 3:1:11) hydrolysis process. The study reported that the extracted CNCs had a high crystallinity index (55.78% ± 7.82%), high aspect ratio, and good thermal stability, with a diameter of 3 to 35 nm [9]. 

Xiong et al. [11] isolated spherical CNCs from MCC derived from waste cotton fabrics using the acid hydrolysis process assisted by ultrasonic treatment. The study reported that waste cotton fabrics are promising raw materials for the isolation of CNCs due to their high cellulose content (94%). Zhong et al. [20] isolated CNCs from recycled indigo-dyed denim fabrics using sulfuric acid hydrolysis for use as a reinforcing agent in polyvinyl alcohol film. It was found that the use of CNCs as a reinforcing agent in polyvinyl alcohol film enhanced the mechanical properties. Dias Maciel et al. [21] isolated CNCs from unwoven industrial textile cotton waste using sulfuric acid hydrolysis with a varying acid concentration ranging from 50 to 64 wt.% and reaction times of 60 and 75 min. The study reported that the optimal experimental condition of the acid hydrolysis process was the sulfuric acid concentration of 64 wt.% and a 60 min reaction time. The characterization of isolated CNCs showed that the sulfuric acid concentration influences the CNCs’ lengths. Although studies have been conducted on the isolation of CNCs from waste cotton cloths from leftover cotton cloths or cotton fabrics in the textile industry, few studies have been conducted on the isolation of CNCs from waste cotton cloths disposed of in a landfill. The utilization of waste cotton cloths for CNC isolation will provide sensible information on the utilization of waste materials for value-added product production. Additionally, CNC isolation from waste cotton cloths would comply with the 3R (Reduce, Reuse and Recycle) concept of sustainability as it would (i) include the reuse of waste materials, (ii) reduce the municipal waste load disposed of in a landfill, and (iii) recycle waste materials to produce value-added products. 

The acid hydrolysis process is the most promising method for producing CNCs from various lignocellulosic sources [4,9]. H_2_SO_4_ and HCl are the most common inorganic acids used in the acid hydrolysis process to remove amorphous regions for producing CNCs. However, studies have reported that CNCs isolated using HCl hydrolysis had a weak oxidizing ability, low thermal degradation, and poor dispersion ability. Conversely, the acid hydrolysis process using H_2_SO_4_ provided the most stable cellulose suspension due to the presence of the sulphate group on the surface of the crystallites, and therefore, the CNCs isolated using the H_2_SO_4_ hydrolysis process gained a better mechanical and thermal stability [4,22]. In the present study, CNCs were isolated from waste cotton cloths using the H_2_SO_4_ hydrolysis process, assisted by ultrasonic treatment of the CNC suspension. Several analytical methods, such as field-emission scanning electron microscopy (FE-SEM), energy-filtered transmission electron microscopy (EF-TEM), Fourier transform infrared spectroscopy (FTIR), X-ray diffraction (XRD), thermal thermogravimetric analysis (TGA), and differential scanning calorimetry (DSC), were utilized to determine the morphological, physicochemical, and thermal properties of the isolated CNCs. The findings of the present study will provide a theoretical foundation for the utilization of waste cotton cloths as promising raw materials for the isolation of CNCs and utilization in the production of value-added products.

## 2. Materials and Methods

### 2.1. Sample Collection and Preparation

Ten pieces of waste cotton cloth (3 kg) was collected from the Pulau Burung Landfill Site, Nibong Tebal, Penang, Malaysia. The collected clothes were sterilized and cleaned with supercritical carbon dioxide (scCO_2_) technology at a pressure of 20 MPa and the temperature of 60 °C for a 1 h sterilizing time to remove pathogenic microorganisms, dirt, and impurities [23]. Subsequently, the sterilized clothes were cut manually into small pieces (approx. 2 mm × 2 mm), and stored at 4 °C prior to further use.

### 2.2. Isolation of CNCs

**Figure 1:**
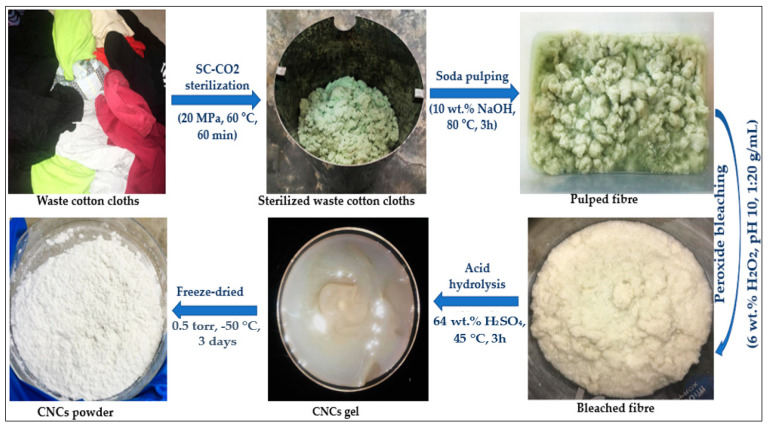
Schematic diagram for the isolation of cellulose nanocrystals (CNCs) from waste cotton cloths.

The isolation of CNCs from the sterilized waste cotton cloths was conducted by alkali pulping, bleaching, and the acid hydrolysis process, as presented in Figure 1. The pulping process was conducted to remove lignin by placing the sterilized waste cotton cloths in 10 wt.% sodium hydroxide (NaOH) solution at a ratio of 1:20. The mixture was then heated at 80 °C for 3 h. After pulping, the pulped fiber was separated by filtration and washed with deionized water until a neutral pH was achieved. Subsequently, the bleaching process was conducted to remove dye and color from the pulped fiber using 6 wt.% hydrogen peroxide (H_2_O_2_) solution at pH 10, with a solid to liquid ratio of 1:20 (g/mL). The pH was adjusted using concentrate NaOH solution. After bleaching, the fiber was washed with deionized water three times and the degreasing fiber (almost 100% cotton) was utilized for the isolation of CNCs. 

The CNCs were obtained from bleached waste cotton cloths fiber by hydrolyzing them in 200 mL of 64 wt.% H_2_SO_4_ solution at 45 ^°^C, with a solid to liquid ratio of 1:10 (g/mL), for the hydrolysis reaction time of 1 h. The hydrolysis reaction was terminated using 400 mL of cool distilled water. The diluted suspension was then centrifuged at 10,000 rpm for 10 min to obtained precipitate, followed by washing with distilled water. This process was repeated until the pH of the suspension was reduced to pH 5 [24]. The washed CNC precipitate was placed in a cellulose membrane dialysis tube, both ends of the dialysis tube were tied with thread, and the sample was dialyzed by changing distilled water until a neutralized pH of 7 was obtained. Subsequently, the CNC suspension was homogenized using a homogenizer (T25 digital Ultra Turrax, IKA, Munich, Germany) at a speed of 5 m/s for 5 min, followed by ultra-sonication at a frequency of 80 kW and amplitude of 0.3% for 15 min in an ice bath, in order to maintain the temperature to prevent it from overheating. The suspension was then freeze-dried (lyophilized) at a temperature of −50 °C and vacuum pressure of 0.5 torr for three days [25]. Finally, dry CNC powder was collected for characterization. The yields of pulped fiber, bleached fiber, and CNC were determined using the following equations [26]:

(1) $${\mathrm{Yield}}_{\mathrm{pulp}\;\mathrm{fiber}}(\%)=\frac{\mathrm{Weight}\;\mathrm{of}\;\mathrm{pulped}\;\mathrm{fiber}}{\mathrm{Weight}\;\mathrm{of}\;\mathrm{the}\;\mathrm{waste}\;\mathrm{cotton}\;\mathrm{cloths}}\times 100\;$$

(2) $${\mathrm{Yield}}_{\mathrm{bleached}\;\mathrm{fiber}}(\%)=\frac{\mathrm{Weight}\;\mathrm{of}\;\mathrm{bleached}\;\mathrm{fiber}}{\mathrm{Weight}\;\mathrm{of}\;\mathrm{pulped}\;\mathrm{fiber}}\times 100$$

(3) $${\mathrm{Yield}}_{\mathrm{CNCs}}(\%)=\frac{\mathrm{Weight}\;\mathrm{of}\;\mathrm{CNCs}}{\mathrm{Weight}\;\mathrm{of}\;\mathrm{bleached}\;\mathrm{fiber}}\times 100\;$$

### 2.3. Morphological Analysis

The surface morphologies of waste cotton cloths, pulped fiber, bleached fiber, and isolated CNCs were observed using field-emission scanning electron microscopy (FE-SEM) (Quanta FEG 650, FEI, Waltham, MA, USA) at 1500 magnification and an accelerating voltage of 5 kV. The images and dimensions of isolated CNCs were observed using energy-filtered transmission electron microscopy (TEM) (TEM Libra 120, Carl Zeiss AG, Oberkochen, Germany) at 12,500 magnification. The isolated CNC suspension was ultra-sonicated in the cool water bath at a frequency of 80 kW and an amplitude of 0.3% for 15 min. A small droplet (1–2 drops) of CNC suspension was deposited on a poly-carbon film supported on a copper grid and air-dried for 5 min. The grid was stained with a small droplet (1–2 drops) of the negative stain of Uranyl acetate solution and air-dried for 5 min before viewing it at an accelerating voltage of 80 kV. The length and diameter of isolated CNC particles were measured using image analysis software (Fiji ImageJ-Version 1.53e for Windows 64-bit, Madison, WI, USA).

### 2.4. ATR-FTIR Analysis

The samples (5–8 mg each) were dried in an oven at 50 °C overnight before being analyzed with Fourier transform infrared spectroscopy (FTIR) (IRPrestige-21, Shimadzu, Kyoto, Japan) equipped with an attenuated total reflectance (ATR) accessory (ATR-8000A, Shimadzu, Kyoto, Japan). The spectra determined the functional groups of cellulose present in waste cotton cloths, pulped fiber, bleached fiber, and isolated CNCs. The spectra were recorded at the wavenumber of 600–4000 cm^−1^ at the spectral resolution of 4 cm^−1^ with the scanning speed of 20 mm/s and 32 scans.

### 2.5. XRD Analysis

X-ray diffraction (XRD) patterns of waste cotton cloths, pulped fiber, bleached fiber, and isolated CNCs were analyzed using an X-diffractometer (XRD-6000, Shimadzu, Kyoto, Japan), operated at a voltage of 40 kV and current of 40 mA, with the scanning rate of 4 s/step, step size of 0.05°/min and scattering 2θ angles from 10 to 40° using a Cu-Kα radiation source (λ = 1.5406 Å). The percentage crystallinity index (CrI) of the samples was determined by referring to the diffraction intensity of the crystalline and amorphous region, calculated by the peak height method using the equation below [27]:

(4) $$\mathrm{CrI}(\%)=\frac{I_{200}-I_{am}}{I_{200}}\times 100,$$

where *I*_200_ is the maximum diffraction intensity associated with surface areas of crystalline cellulose, and *I_am_* is the diffraction intensity of an amorphous cellulose fraction.

### 2.6. Thermal Properties Analysis

The thermal properties of waste cotton cloths, pulped fiber, bleached fiber, and isolated CNCs were determined using thermogravimetric analysis (TGA/DSC 1 HT, Mettler-Toledo, Colombus, OH, USA). About 8 mg of sample was placed in an aluminium pan, and the sample was then heated from room temperature (28 ± 1 °C) to 800 °C with a heating rate of 10 °C/min and nitrogen gas flow of 50 mL/min. The thermal stability of waste cotton cloths, pulped fiber, bleached fiber, and isolated CNCs was determined using differential scanning calorimetry (DSC) (DSC Q200, TA Instruments, Lukens Dr, New Castle, DE, USA). The changes in weight and heat flow were analyzed.

### 2.7. Physical Properties Analysis

#### 2.7.1. Moisture Content

The moisture content of the sample was determined using the Technical Association of the Pulp and Paper Industry(TAPPI) method [28]. About 5 g of sample was taken randomly and weighed to the nearest 0.001 g in a tared aluminium foil in a moisture analyzer. Subsequently, the lid of the moisture analyzer was closed and heated to 105 °C for 5 min. The reading in percentage obtained from the moisture analyzer was taken as the percentage of moisture content.

#### 2.7.2. Cellulose Content

The determination of the holocellulose in the experiment was conducted following the chlorination method and Wise method [29], carried out in a fume cupboard due to the toxicity of chlorine dioxin. In total, 2 g of oven-dried extractive-free sample was weighed in a 250 mL Erlenmeyer flask. Then, 100 mL of distilled water was added, followed by 1.5 g sodium chlorite (NaClO_3_) and 5 mL of 10% acetic acid (CH_3_COOH), before being placed in a water bath at 70 °C for 30 min with frequent stirring. Following this, the addition of 1.5 g sodium chlorite (NaClO_3_) and 5 mL of 10% acetic acid (CH_3_COOH) was repeated another three times at 30 min intervals in a water bath at 70 °C. The sample was allowed to cool to 10 °C, was filtered in a weighed filtering crucible (2G2), and was washed with cool distilled water (4–8 °C). Subsequently, the sample was washed with acetone and allowed to air-dry. The residue was transferred into desiccators and weighed at daily intervals, until the sample reached a constant weight [30]. 

The α-cellulose content was determined using Japanese Standard Method JIS 8101- Determination of the alpha-cellulose content [31]. In total, 1 g of oven-dried holocellulose was weighed in a 100 mL Erlenmeyer flask and placed in a water bath at 25 °C for 30 min. Then, 10 mL of 17.5% sodium hydroxide (NaOH) was added into the flask and left for 3.5 min with occasional stirring. Subsequently, the mixture was smashed and stirred with a glass rod for 5 min, and the surface of the mixture was then flattened for 30 s. The flattened mixture was allowed to stand in a water bath for 20 min. Next, 10 mL of distilled water was added to the mixture. Immediately after this, the mixture was stirred thoroughly for 1 min and left for 5 min. The mixture was transferred to a weighed filtering crucible (2G2), rinsed with distilled water, and then rinsed using 8 mL of 10% acetic acid (CH_3_COOH) for 5 min. Following this, the sample was rinsed with 200 mL boiled distilled water, and the filtrate was dried in an oven at 105 °C until it had reached a constant weight, while the filtrate was kept for the further experiment of the Beta- and Gamma-cellulose content. The weight was retaken after the sample had been cooled for 20 min in desiccators.

The β- and γ-cellulose content was determined according to Japanese Standard Method JIS 8101- Determination of the alpha-cellulose content [31,32]. Filter paper and a weighing bottle were dried in an oven at 105 °C. The filter paper was then kept in a weighing bottle and cooled inside desiccators. The filtrate from the previous experiment on the α-cellulose content was poured into a 250 mL beaker, and 16 mL of 30% acetic acid (CH_3_COOH) was added. The mixture was boiled slowly to around 100 °C, and β-cellulose was flocculated in the mixture. The mixture was cooled for 20 min at room temperature, followed by filtering through the weighed filter paper, and washed with lukewarm distilled water. The sample was dried at 105 °C until reaching a constant weight. The γ- cellulose content was calculated as below [33,34]:

(5) $$\gamma_{\mathrm{Cellulose}}(\%)=100-(\alpha_{\mathrm{Cellulose}}-\beta_{\mathrm{Cellulose}})\;.$$

#### 2.7.3. Acid Insoluble Lignin

Acid insoluble lignin (Klason lignin) was determined using the method of TAPPI T222 om-02- Acid insoluble lignin in wood and pulp [33]. In total, 1 g of oven-dried extractive-free sample was weighed. Then, 15 mL of cool (10–15 °C) 72% H_2_SO_4_ was slowly added with constant stirring using a magnetic stirrer at 25 °C for 2 h to ensure that the sample and acid were thoroughly mixed. The mixture was diluted to an acid concentration of 3% in a 1 L Erlenmeyer flask using hot distilled water and boiled gently for 4 h. The volume of the mixture was maintained continuously by the frequent addition of hot distilled water. Subsequently, the insoluble lignin was allowed to stand overnight to settle, before being filtered in a weighed filtering crucible. The residue was dried in an oven at 105 °C until a constant weight was obtained. 

#### 2.7.4. Kappa Number

The Kappa numbers of the waste cotton cloths, pulped fiber, bleached fiber, and CNCs were determined using the TAPPI T 236 om-99- Kappa number of pulp [34]. A 1 g oven-dried sample was weighed and disintegrated in 100 mL of distilled water using a blender until all fiber bundles were dispersed. The disintegrated sample was transferred into a 500 mL beaker and blender and rinsed with 95 mL of distilled water. The beaker was placed on a magnetic stirrer, and the suspension was continuously stirred to introduce air into the mixture. Then, 25 mL of 0.1 N potassium permanganate (KMnO_4_) and 25 mL of 4 N H_2_SO_4_ were quickly added to the disintegrated sample, followed by 5 mL distilled water, and simultaneously stirred for 10 min. The total volume of the mixture obtained should have been 250 mL. After 10 min, 5 mL of 1 N potassium iodide (KI) was added to stop the reaction and free iodine solution was titrated with 0.1 N sodium thiosulfate (Na_2_S_2_O_3_) solution. When the mixture turned developed a light-yellow colour, a few drops of the starch indicator were added, and a blue color appeared. The titration continued slowly until no color was observed. A blank determination was carried out using the same method, but without a sample [34]. Meanwhile, the Kappa number of all samples was calculated as follows:

(6) $$\rho =\frac{b-a}{N}\times 100,$$

(7) $$K=\frac{\rho f}{w}\;,$$

where *K* is the Kappa number, *f* is a factor for correction to a 50% permanganate consumption, w is the weight of moisture-free pulp in the specimen (g), p is the amount of 0.1 N permanganate consumed by the test specimen (mL), b is the amount of thiosulfate consumed in the blank determination (mL), a is the amount of thiosulfate consumed by the test specimen (mL), and N is the normality of the thiosulfate.

#### 2.7.5. Whiteness

The percentage whiteness of pulped fiber, bleached fiber, and CNCs was determined using a calorimeter (Minolta Spectrophotometer CM-3500d with Spectra Magic Software, Konica Minolta, Tokyo, Japan) by following TAPPI T 452 om-98- Brightness of pulp, paper, and paperboard (directional reflectance at 457 nm) [35]. The sample was placed in a Petri dish in the calorimeter and the whiteness reading was recorded.

#### 2.7.6. Production Cost

The CNC production cost from the waste cotton cloths was estimated by considering the cost of sample collection, chemical, and reagent for calculating the cost for the isolation of CNCs from waste cotton cloths, energy consumption, and labor cost.

### 2.8. Zeta Potential and Dynamic Light Scattering (DLS) Using Zetasizer

The hydrodynamic size measurement of isolated CNCs was conducted by dynamic light scattering (DLS), measured using Zetasizer Nano-ZS equipment (Malvern, Worcestershire, UK). CNCs were diluted to 0.1 wt.% in deionized water, and the aqueous suspension was then filtered using a 0.45 um syringe filter and ultrasonic at 80%, with a frequency of 0.3 for 1 h in an ice bath. Standard quartz cuvettes were used for the measurement and the experiments were repeated three times at room temperature. The average particle size and size distribution of isolated CNCs in aqueous solution were determined. 

## 3. Results and Discussion

### 3.1. Morphological Analysis

**Figure 2:**
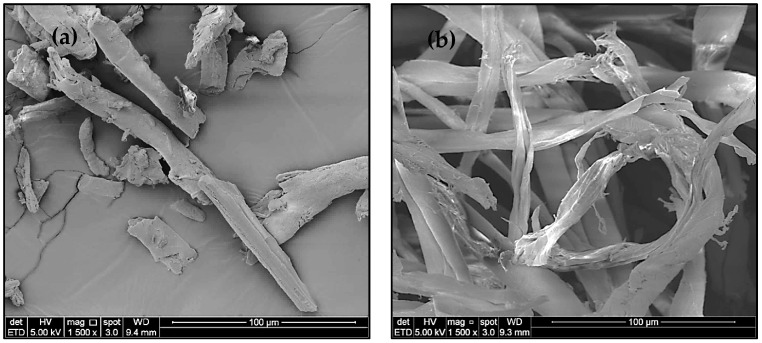

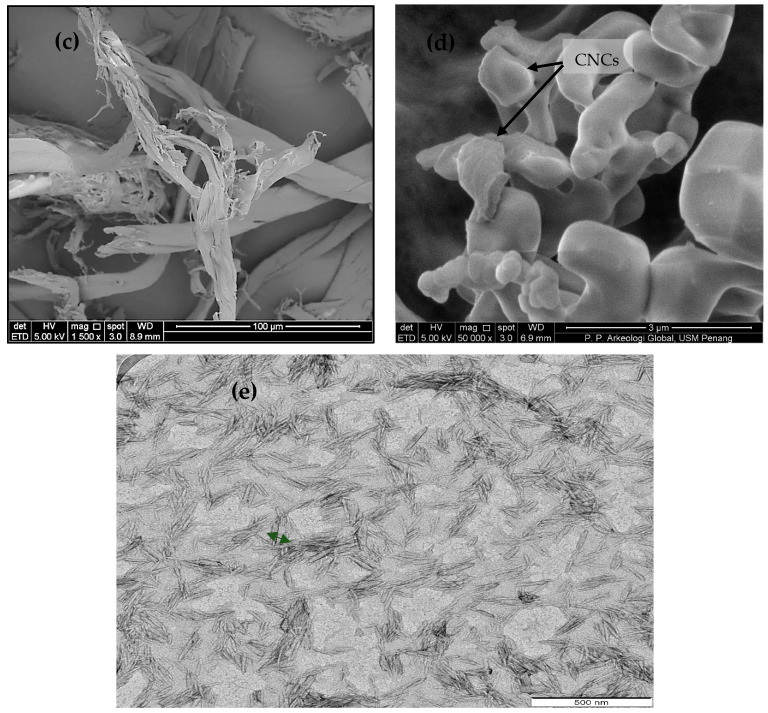
Field emission scanning electron microscope images of (**a**) waste cotton cloths, (**b**) pulped fiber, (**c**) bleached fiber, and (**d**) isolated CNCs, and (**e**) an energy-filtered transmission electron microscopy image of CNCs.

The surface morphology of waste cotton cloths, pulped fiber, bleached fiber, and isolated CNCs was analyzed using a field emission scanning electron microscope image (FE-SEM), as shown in Figure 2. The waste cotton cloths were spun and dyed using physical and chemical treatment, being used repeatedly, and underwent numerous laundry, drying, and ironing processes. It was found that the surface of the waste cotton cloths was flat and exhibited a spiral, with a length, diameter, and aspect ratio range of 50–100 µm, 10–30 µm, and 5–10, respectively. Meanwhile, the surface of the pulped and bleached fibers was swollen due to soda pulping and peroxide bleaching. These fibers had a rough surface, while the outer layer was disrupted and cracked along the inner structure, exposing the cellulose fibril strand. This can be attributed to the effective removal of hemicellulose, lignin, waxes, and other impurities during soda pulping and peroxide bleaching, where these components are supposed to provide rigidity, permeability, and protection of the fiber structure in waste cotton cloths. The pulped fiber average length and diameter were 150 and 15 µm, respectively. Furthermore, the bleached fiber average length and diameter were 100 and 10 µm, respectively. After acid hydrolysis, the cellulose fiber became shorter to produce a nano-size cellulose fiber. This happened because the acid penetrated the amorphous region of cellulose and cleaved the β-1,4-linkage between cellulose repeating units [9,22,36]. Therefore, it left the only crystalline region and broke the cellulose fiber into shorter cellulose nanocrystals. The ordered crystalline arrangements appeared due to the formation of inter-and intra-molecular H-bonding between the hydroxyl groups. The H-bonding hindered the free movement of cellulose chains and aligned in an orderly manner. Cellulose fiber from waste cotton cloths was successfully defibrillated to CNCs with rod-like structures. However, the shape, length, and diameter of isolated CNCs were unable to be determined using FE-SEM.

The surface morphology the isolated CNCs was observed using energy-filtered transmission electron microscopy (EF-TEM), as shown in Figure 2e. It was found that the cellulose fiber from waste cotton cloths effectively defibrillated to CNCs using the acid hydrolysis process, assisted by the ultrasonication process. The approximate length, diameter, and aspect ratio of CNCs were determined to be 10–30 and 2–6 nm and 12–15, respectively. Therefore, the CNCs extracted from the waste cotton cloths could be utilized as an adsorbent or reinforcing material in composite materials because of their smaller particle size and high aspect ratio [4,9]. Wang et al. [9] isolated CNCs from old cotton cloths (used bed sheet) using a mixed acid hydrolysis method. The length, diameter, and aspect ratio of the isolated CNCs were 28–470 nm, 3-35 nm, and 17 ± 15, respectively. The study reported that the CNCs isolated from old cotton cloths had the potential to be used as a reinforcing agent for increasing the physical properties of the composite materials. Huang et al. [4] isolated CNCs from textile waste using acid hydrolysis and a three-step oxidation process and utilized isolated CNCs as a reinforcing agent in soy protein film. The aspect ratios of the isolated CNCs using acid hydrolysis and the three-step oxidation process were 10 ± 3 and 17 ± 13, respectively. The study reported that the incorporation of CNCs from textile waste significantly increased the tensile strength, water vapor barrier properties, and Young’s modulus. Table 1 shows the size and percentage yield of CNCs isolated from the various cellulosic sources. It was found that the length (10–30 nm) and diameter (2–6 nm) of the isolated CNCs from waste cotton cloths collected from the landfill had lower values than those for CNCs isolated from old bedsheet, degreasing cotton, cotton, textile waste, and commercial cotton fiber. Additionally, the obtained CNCs’ yield (65.40 ± 2.1%) was higher than that of CNCs isolated from old bedsheet, degreasing cotton, cotton, textile waste, and commercial cotton fiber. The variation of the results obtained in the present study might be due to the application of scCO_2_ sterilization technology for cleaning the waste cotton cloths. The scCO_2_ is a waterless cleaning technology, which does not degrade the fiber quality due to its low operating temperature and moderate pressure [37].

### 3.2. ATR-FTIR Analysis

**Figure 3:**
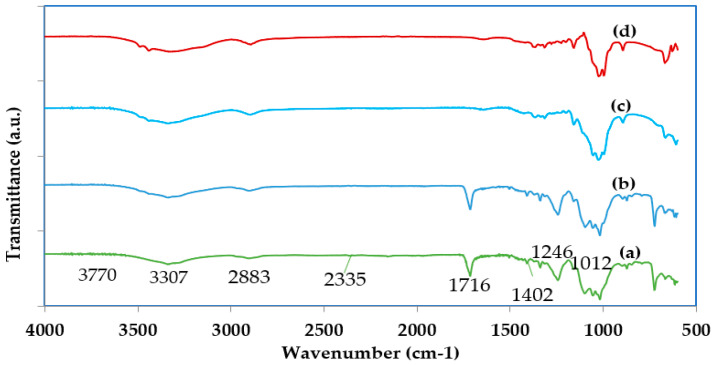
Attenuated total reflection-Fourier transform-infrared spectroscopy (ATR-FTIR) spectra of (**a**) waste cotton cloths, (**b**) pulped fiber, (**c**) bleached fiber, and (**d**) isolated CNCs.

Figure 3 shows the ATR-FTIR spectra of waste cotton cloths, pulped fiber, bleached fiber, and CNCs. The stretching vibration of O–H and asymmetric stretching vibration of the C–H bond appeared at 3340 and 2900 cm^−1^, respectively. The peak observed at 1640 cm^−1^ is related to the absorbed water in the cellulose fiber [9]. Although all samples were oven-dried prior to analyses, it is impossible to completely dry the cellulose fiber from waste cotton cloths because of its strong interaction with moisture. The broad peak at 3336 cm^−1^ is related to the stretching vibration of OH groups and the inter-chain hydrogen bonds. The peak at 2900 cm^−1^ can be attributed to the stretching vibration of C–H, and the peaks at 1427, 1371, and 1315 cm^−1^ can be attributed to the bending of C–H, CH_2_, and OH, respectively, which are typical for polysaccharides. The peak at 1160 cm^−1^ is characteristic of the asymmetric vibration of (C–O–C), and those at 1055 and 1031 cm^−1^ are associated with C–O–C pyranose ring (antisymmetric in phase ring) stretching vibration. The peak at 893 cm^−1^ is characteristic of cellulose with β-glycoside bonds of the glucose ring. The characteristic peaks of cellulose appeared at 3290 and 3336 cm^−1^, and the peak at 1427 cm^−1^ is attributable to the crystalline absorption. The absorbance ratio of the bands at 1427 and 893 cm^−1^ adopted as the crystallinity index is closely related to the portion of the cellulose structure. A new peak of the isolated CNCs was observed at 1616 cm^−1^, which was assigned to OSO_3_ or oxidation of the C–OH groups [42]. The adsorption peak in the ATR-FTIR spectra of waste cotton cloths (Figure 3a) and pulped fiber (Figure 3b) occurred at 1716 and 1242 cm^−1^, respectively. The adsorption peak at 1716 cm^−1^ can be assigned to the C=C in-plane aromatic skeleton vibrations of lignin. Moreover, the adsorption peak at 1242 cm^−1^ can be attributed to the C–O–C stretching for the ether linkage lignin. However, the absence of these adsorption peaks in the ATR-FTIR spectra of bleached fiber (Figure 3c) and CNCs (Figure 3d) revealed that bleaching and the acid hydrolysis process effectively removed the lignin from the waste cotton cloths [43].

### 3.3. Crystallinity Index Using XRD

**Figure 4:**
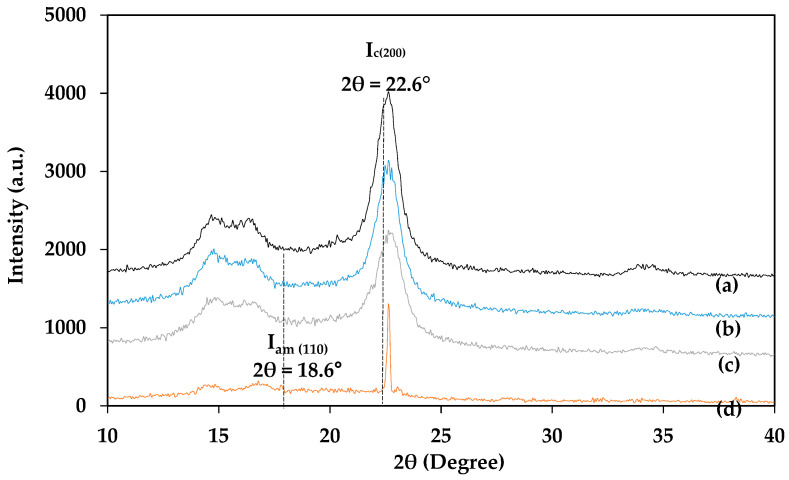
X-ray diffraction (XRD) patterns of (**a**) bleached fiber, (**b**) pulped fiber, (**c**) waste cotton cloths, and (**d**) isolated CNCs.

X-ray diffraction (XRD) is a useful tool for determination of the cellulose crystalline structure. Figure 4 shows the XRD patterns of waste cotton cloths, pulped fiber, bleached fiber, and isolated CNCs. It was found that the diffractions of waste cotton cloths, pulped fiber, bleached fiber, and CNCs exhibited similar diffraction peaks at 14.8°, 14.18.6°, 22.6°, and 34.6°, corresponding to (110), (110), (200), and (004) cellulose crystallography planes of the cellulose I lattice, respectively [9,43]. The observation of similar diffraction peaks in the XRD patterns of waste cotton cloths, pulped fiber, bleached fiber, and isolated CNCs indicates that CNC isolation processes such as alkaline pulping, bleaching, and acid hydrolysis did not affect the cellulose structure. Based on the intensity of the peaks of waste cotton cloths, pulped fiber, bleached fiber, and isolated CNCs, it can be estimated that the degrees of cellulose crystallinity of pulped fiber, bleached fiber, and isolated CNCs were higher than that in waste cotton cloths. The percentage crystallinity indexes of waste cotton cloths, pulped fiber, bleached fiber, and isolated CNCs calculated using the Segal equation (Equation (4)) were 68.29%, 75.21%, 78.98%, and 83.46%, respectively. The increases of the crystalline index in pulped fiber, bleached fiber, and isolated CNCs can be attributed to the removal of the amorphous region of cotton fibers of waste cotton cloths with alkaline pulping, bleaching, and the acid hydrolysis process. The highest percentage crystallinity index was obtained in CNCs because of the penetration of H_2_SO_4_ solution into the amorphous region of cellulose fiber, which broke down the glycosidic bond and released the individual crystallites [44]. The finding was found to be consistent with other studies in the literature. Wang et al. [9] found a higher crystalline index in the isolated CNCs than the parent materials. Huang et al. [4] isolated CNCs from textile waste and observed a remarkable increase of the percentage crystalline index in isolated CNCs (89.88%) compared to textile waste (73.42%). The study reported an increase of the percentage crystalline index in CNCs compared to textile waste because of the removal of the amorphous region of the cotton fiber during the extraction of CNCs with the H_2_SO_4_ solution.

### 3.4. Thermal Stability Analysis

**Figure 5:**
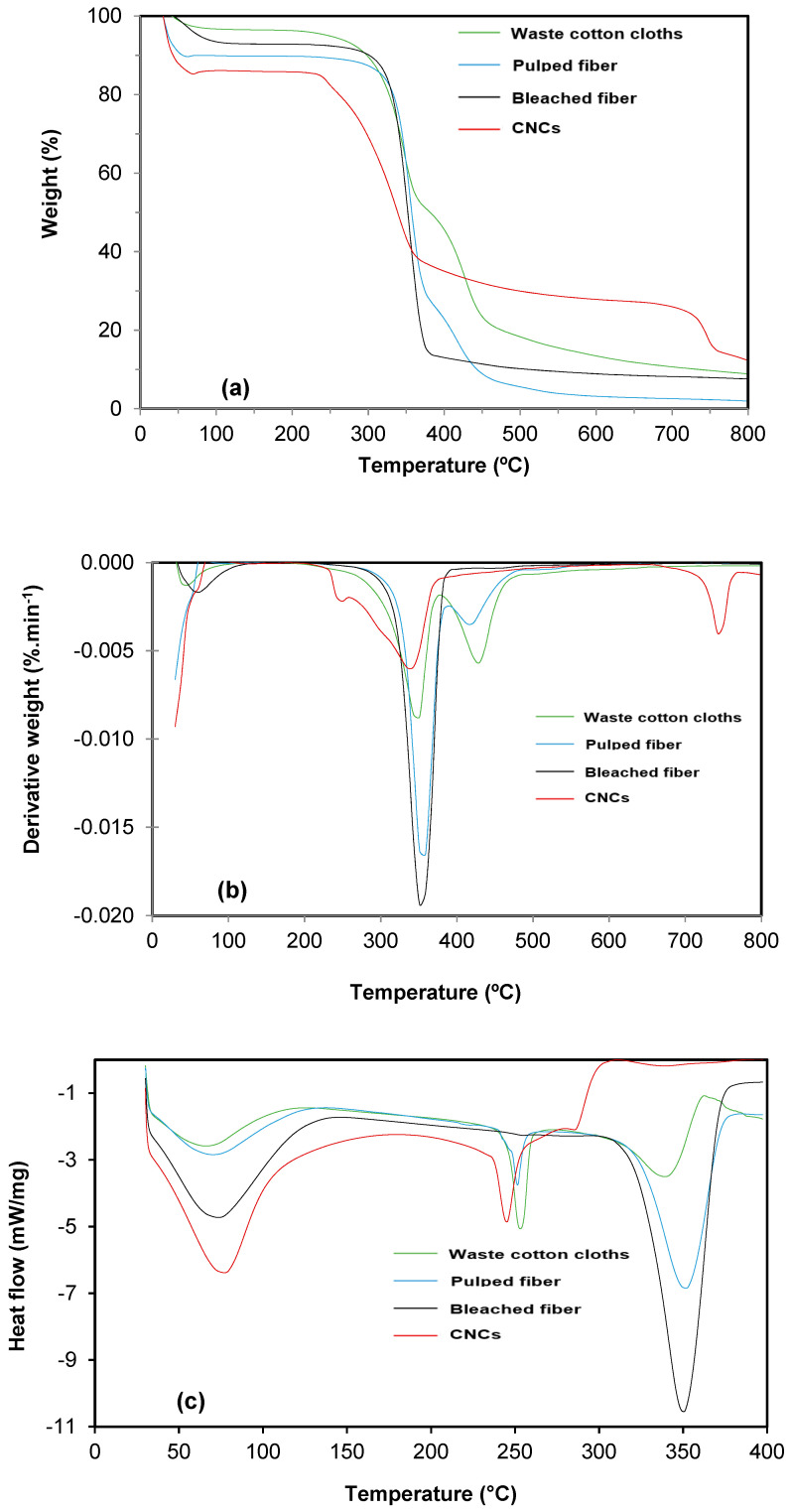
Thermal stability analyses of waste cotton cloths, pulped fiber, bleached fiber, and CNCs. (**a**) Thermogravimetric analysis (TGA), (**b**) derivative thermogravimetric analyses (DTGA), and (**c**) differential scanning calorimetry (DSC).

Thermal stability analyses of waste cotton cloths, pulped fiber, bleached fiber, and CNCs were conducted using thermogravimetric analyses (TGA), derivative thermogravimetric analyses (DTGA), and differential scanning calorimetry (DSC), as shown in Figure 5. Negligible weight loss was found for waste cotton cloths, pulped fiber, bleached fiber, and CNCs below 200 °C. This happened due to the vaporization of the moisture present in the waste cotton cloths, pulped fiber, bleached fiber, and CNCs [12,39]. Additionally, the presence of moisture could be attributed to the hydrophilicity of waste cotton cloths, pulped fiber, bleached fiber, and CNCs [23]. The thermal degradation of samples occurred within the temperature range of 200–400 °C. The maximum thermal degradation temperature (T_max_) for the maximum weight loss of waste cotton cloths, pulped fiber, bleached fiber, and CNCs was 361, 359, 351, and 341 °C, respectively, and the corresponding weight loss was determined to be 79.38%, 82.98%, 86.51%, and 63.57% (Figure 5a,b). It was found that alkaline pulping and the bleaching process had a minimal impact on the thermal stability of pulped fiber and bleached fiber, as the thermal degradation temperatures for the pulped fiber and bleached fiber were almost similar. The results were consistent with the studies reported by Wang et al. [9] and Lamaming et al. [13]. Wang et al. [9] found that the alkali and bleaching treatment of waste cotton cloths had a minimal effect on the thermal stability. Lamaming et al. [13] observed that alkaline pulping and the bleaching process of oil palm trunk fiber had a negligible impact on the thermal stability.

The lowest weight loss of CNCs was obtained at a relatively lower T_max_ temperature than that of pulped and bleached fibers, indicating that the CNCs isolated from the waste cotton cloths using sulfuric acid hydrolysis had the highest thermal stability [4,13,45]. The enhanced thermal stability of CNCs can be attributed to the removal of amorphous compounds from the cellulose chain [9]. The thermal degradation temperature of waste cotton cloths, pulped fiber, bleached fiber, and CNCs leveled off or decreased slowly over 400 °C, which can be ascribed to the carbonization of polysaccharide chains initiated with the cleavage of C–H and C–C bonds.

The DSC curves for waste cotton cloths, pulped fiber, bleached fiber, and isolated CNCs are shown in Figure 5c. It was found that the initial endothermic peak occurred below 100 °C, which was caused by moisture evaporation from waste cotton cloths, pulped fiber, bleached fiber, and CNCs. The endothermic peak was obtained at around 250 °C on the DSC curves of pulped fiber, bleached fiber, and isolated CNCs, which can attributed to the decomposition of cellulose fiber in pulped fiber, bleached fiber, and isolated CNCs. The DSC curve for the waste cotton cloths showed a shoulder peak at around 350 °C. It was found that the height of the shoulder peak decreased with soda pulping and the bleaching process, indicating the removal of lignin and hemicellulose from the waste cotton cloths. However, the shoulder peak disappeared after the acid hydrolysis of bleached fiber, indicating the complete removal of lignin and hemicellulose from the waste cotton cloths [9]. Based on the thermal properties analysis, it can be concluded that CNCs isolated from the waste cotton cloths had a good thermal stability. Therefore, the waste cotton cloths could be utilized to isolate CNCs, which would minimize the municipal waste load dispose of in landfills, minimize the environmental pollution, and enhance the sustainable utilization of waste materials to produce value-added products. 

### 3.5. Physical Properties Analysis

Table 2 shows the physical properties analyses of waste cotton cloths, pulped fiber, bleached fiber, and isolated CNCs. The moisture content of waste cotton cloths, pulped fiber, bleached fiber, and isolated CNCs was 7.4%, 7.3%, 7.2%, and 6.5%, respectively. All samples were kept dry to prevent them from being consumed by a microorganism, such as fungi. The cellulose content of waste cotton cloths, pulped fiber, bleached fiber, and isolated CNCs was 81.38%, 87.38%, 88.13%, and 97.87%, respectively. The increase in cellulose content in treated fiber was due to removing lignin and hemicellulose from the waste cotton cloths with pulping, bleaching, and acid hydrolysis processes [4,22]. Holocellulose consists of cellulose and hemicellulose (γ-cellulose), where cellulose is comprised of α-cellulose and β-cellulose. While the percentage of cellulose content increased with pulping, bleaching, and acid hydrolysis treatment, the percentage of hemicellulose and lignin content were decreased with the processes. The hemicellulose content in waste cotton cloths, pulped fiber, bleached fiber, and isolated CNCs was 14.29%, 6.83%, 4.33%, and 2.10%, respectively. Moreover, the lignin content in waste cotton cloths, pulped fiber, bleached fiber, and isolated CNCs was determined to be 8%, 5.79%, 2.83%, and almost nil, respectively. The Kappa number is an indication of the lignin content or bleachability of pulp, ranging from 1 to 100 [46]. In this study, a decreasing trend of the Kappa number was obtained. The Kappa numbers for waste cotton cloths, pulped fiber, and bleached fiber were 16.81, 14.88, and 13.92, respectively. The ash content, representing an inorganic residue, indicates the incombustible component remaining after a sample was determined from TGA and DSC analyses. However, a small amount of inorganic materials remained in raw waste cloths, which might be due to the presence of polyester fiber in the collected waste cotton cloths from the landfill. The percentage of ash content decreased in pulped fiber, followed by bleached fiber and CNCs. The whiteness of waste cotton cloths, pulped fiber, and bleached fiber increased with the treatment processes applied for the isolation of CNCs from the waste cotton cloths. This is due to the fact that the color and dye were effectively removed from the waste cotton cloths using alkaline pulping and the bleaching process. The estimation of CNCs’ production cost is summarized in Table S1. Approximately, 1 kg of waste cotton cloths was employed for CNCs, which yielded about 654 g of CNCs. The emitted production cost for the per gram CNC production was determined to be USD 0.19. However, the production cost would decrease in large-scale production.

### 3.6. Surface Charge, Particle Size, and Size Distribution Analysis

The Zeta potential is a measure of the magnitude of the electrostatic or charge repulsion/attraction between particles. It is one of the fundamental parameters known to affect the stability. Its measurement provides detailed insights into the causes of dispersion, aggregation, or flocculation. From Zeta potential analysis of the isolated CNCs, the Zeta potential charge was found to be −32.9 mV, with the conductivity of 0.0248 mS/cm. According to reported studies, CNCs obtained by acid hydrolysis with H_2_SO_4_ have negative surface charges due to the addition of sulfate groups on the surface of cellulose during acid hydrolysis, which results in electrostatically stable colloidal aqueous suspensions and prevents the sedimentation and agglomeration of CNCs in aqueous suspension, due to the esterification of surface hydroxyl groups resulting in sulfate groups [47,48]. Surface charge modification by the addition of sulfuric acid during acid hydrolysis (sulphate group) introduces anionic functionality to increase the defibrillation of cellulose based on the principle of electrostatic repulsion between the similar charge of the negative charge (anions). CNCs are negatively charged colloidal particles and are well-known to form highly stable surfactant-free substances [48]. Dynamic light scattering (DLS) is a method which measures the frequency shift of laser light reflected by particles in suspension moving by Brownian motion. The distribution of particles’ speed is converted into the size distribution. From the analysis conducted in this study, it was found that the particle size of isolated CNCs varied from 37.84 to 342 nm, with the average particle size of 109.1 nm. The results varied with the particle size determined from TEM analyses. This might be due to some of CNC particles not having fully dissolved in water, and therefore, the size of CNCs may not have been homogeneous [48].

## 4. Conclusions

In the present study, CNCs were successfully isolated from waste cotton cloths using the acid hydrolysis process. The morphological analyses revealed that the isolated CNCs had a needle-like crystalline structure with a length, diameter, and aspect ratio of 10–30 nm, 2–6 nm, and 5–15, respectively. The ATR-FTIR spectra analyses showed that the resultant CNCs were cellulose species. The XRD analyses revealed that the percentage crystallinity index was enhanced with acid hydrolyses, and the highest percentage crystallinity index obtained for the isolated CNCs was 83.46%. Thermal stability analyses showed that the CNCs isolated from waste cotton cloths had a good thermal stability, and have the potential to be utilized as filler or reinforcing agents in a composite material. The findings of the present study revealed that waste cotton cloths could be utilized as raw materials for the production of CNCs. This innovative utilization of waste cotton cloths would lead to reducing the municipal waste load disposed in a landfill, minimizing the environmental pollution, and enhancing the sustainable utilization of waste materials for producing value-added products.

## Figures and Tables

**Table 1:** Size and percentage yield of CNCs isolated from the various raw materials.

Raw Materials	Length (nm)	Diameter (nm)	Yield CNC (%)	Methods	References
Commercial cotton balls	150 ± 50	14 ± 5	60	H_2_SO_4_ hydrolysis	[38]
Cotton	450	25	77	H_2_SO_4_ hydrolysis	[16]
Cotton	287.24 ± 79.75	29.69 ± 5.07	51	H_2_SO_4_ hydrolysis	[39]
Cotton	120.27 ± 36.25	40.74 ± 7.59	22	Enzymatic hydrolysis	[39]
Cotton fabrics	76–159	14.2–15	30–35	H_2_SO_4_ hydrolysis	[40]
Cotton linters	161–193	10–13	74–80	H_2_SO_4_ hydrolysis	[41]
Degreasing cotton	17–230	2–25	52.4 ± 1.5	Mixed H_2_SO_4_ and HCl hydrolysis	[9]
Old sheet bed	28–470	3–35	46.7 ± 1.8	Mixed H_2_SO_4_ and HCl hydrolysis	[9]
Textile waste from factory	97.25 ± 25.18	5.69 ± 2.08	60.41	Oxidation	[4]
Waste cotton fabrics	5–100	10–65	21.5	H_2_SO_4_ hydrolysis and ultrasonication	[11]
Waste cloths from landfill	10–30	2–6	65.40 ± 2.1	H_2_SO_4_ hydrolysis and ultrasonication	Present study

**Table 2:** Physical properties analysis of waste cotton cloths, pulped fiber, bleached fiber, and isolated CNCs.

Properties	Waste Cotton Cloths	Pulped Fiber	Bleached Fiber	CNCs
Yield (%)	-	80.39 ± 1.0	86.02 ± 1.4	65.40 ± 1.3
Moisture content (%)	7.4 ± 0.03	7.3 ± 0.17	7.2 ± 0.13	6.5 ± 0.13
Cellulose yield (%)	81.38 ± 0.60	87.38 ± 0.62	88.13 ± 0.50	97.87 ± 0.19
α-cellulose (%)	80.39 ± 0.13	86.02 ± 0.24	86.66 ± 0.25	96.82 ± 0.05
β-cellulose (%)	0.99 ± 0.14	1.36 ± 0.14	1.47 ± 0.12	1.05 ± 0.06
Hemicellulose (γ_cellulose_) (%)	14.29 ± 0.33	6.83 ± 0.24	4.33 ± 0.13	2.10 ± 0.08
Klason lignin (%)	8.00 ± 0.52	5.79 ± 0.63	2.83 ± 0.74	ND
Kappa number	16.81 ± 0.97	14.88 ± 0.96	13.92 ± 0.96	ND
Ash content (%)	9.12 ± 0.87	7.71 ± 0.92	7.17 ± 0.86	3.94 ± 0.97
Whiteness (%)	56 ± 2	62 ± 3	86 ± 2	82 ± 4
Production cost (USD/g)	-	-	-	0.19

ND: Not detected.

## Data Availability

The data presented in this study are available on request from the corresponding author.

## Supplementary Materials

The following are available online at https://www.mdpi.com/2073-4360/13/4/626/s1.
