# Supplementary material for: Recycling Waste Cotton Cloths for the Isolation of Cellulose Nanocrystals: A Sustainable Approach
Source: Polymers (Basel). 2021 Feb 19;13(4):626. doi: 10.3390/polym13040626 (PMC7922772; doi:10.3390/polym13040626)
Supplement: Supplementary file 1 [file polymers-13-00626-s001.pdf]

Table S1. Estimated cost production of CNC from Waste cotton cloths.

Processes	Amount	Price/unit (RM)	Estimated cost (RM)
Sample collection			
Waste cotton cloths	1 kg	FREE	0
Transportation (two-way)	100 km	0.79/km	79.00
Sterilization			
CO_2 tank	7.8 L (1 h)	2.00/L	15.60
Power consumption (1 hr)	660 W (1 h)	[1] 0.3945/kWh	0.26
Soda pulping			
Sodium hydroxide (NaOH)	2 kg	42.00/kg	84.00
Water	20 L	0.22/1,000L	4.40
Water bath power (3 hr)	1,800 W (1 hr)	0.3945/kWh	0.71
Bleaching			
Hydrogen peroxide (H_2O_2)	0.907 L	64.00/L	58.05
Water	16.9 L	0.22/1,000 L	3.72
Water bath (3 hr)	220 W (1 h)	0.3945/kWh	0.06
Acid hydrolysis			
Sulfuric acid (H_2SO_4)	3.892 L	90.00/2.5 L	140.11
Water	3.8 L	0.22/1,000 L	0.84
Water bath (3 hr)	220 W (1 h)	0.3945/kWh	0.06
Freeze dryer power (72 h)	1,000 W (1 h)	0.3945/kWh	28.40
Labour cost (1 person)	2 h/day (5 days)	8.00/h	80.00
		**TOTAL**	495.18

[1]Malaysia feed-in tariff (FiT) rates system
